# Retraction Note: LINC01296/miR-26a/GALNT3 axis contributes to colorectal cancer progression by regulating O-glycosylated MUC1 via PI3K/AKT pathway

**DOI:** 10.1186/s13046-026-03700-y

**Published:** 2026-03-27

**Authors:** Bing Liu, Shimeng Pan, Yang Xiao, Qianqian Liu, Jingchao Xu, Li Jia

**Affiliations:** College of Laboratory Medicine, Dalian Medical University, 9 Lushunnan Road Xiduan, Dalian, Liaoning Province 116044 China

**Retraction Note: J Exp Clin Cancer Res 37**,** 31 (2018)**

**https://doi.org/10.1186/s13046-018-0994-x**

The Editor-in-Chief has retracted this article. Concerns were raised regarding a number of figures. Although some of these concerns were addressed with a correction, further concerns were subsequently raised that further undermined the veracity of the article. The image concerns were as follows:

- Figure 5C panel 7 and 5G panel 2: these panels appear to overlap; a correction was issued for Figure 5G panel 2.
- Figure 3J panel 6 and Figure 5C panel 4: these panels appear to overlap; a correction was issued for Figure 5C panel 4.
- Figure 5C panel 8 of this article and Figure 6D panel 7 of [1]: these panels appear to overlap; a correction was issued for Figure 6D panel 7 of [1].
- Figure 6E VVA-binding MUC1 panel of this article and Figure 5D CyclinD1 panel of [2]: these panels appear to overlap; a correction was issued for Figure 5D CyclinD1 panel of [2].
- Figure 7B panel 23 of this article and Figure 3I panel 4 of [3]: these panels appear to overlap; [3] was a previously published article that has since been retracted.

The authors were unable to provide a sufficient response or sufficient raw data to resolve the concerns raised. The Editor-in-Chief therefore no longer has confidence in the reliability of the data reported in the article.

Author Li Jia has stated on behalf of all authors that they disagree with this retraction.
